# On the Chemical Analysis of Vegetables

**Published:** 1801-04

**Authors:** 

**Affiliations:** Berlin


### *On the Chemical Analysis of Vegetables. By Dr.* HERMBSTAEDT, *of Berlin.*

[ Continued from Vol. III. pp. 373. ]

### *On the Separation of sebaceous Matter from Vegetables.*

THE febaceous matter appears in vegetables in different forms, viz. as a fluid oil, as a fubftance of butyraceous confiftence, and as a firm matter like tallow. As a part, mixed in them, it is fo intimately united with gum, mucilaginous matter and water, that it cannot be eafily difcovered. But when thofe fubftances are reduced into fmall particles by comminution, and by continually ftirring them, kept on a gentle fire till the water evaporates, the gum and mucilage hardens, and the febaceous matter may then, in a pure ftate, be feparated by a warm expreffion, and its weight alfo determined,* after having previoufly freed it from all adherent heterogeneous particles.

* As it is very difficult, if not impoffible, to obtain all febaceous matter by repeated expreffions, this circumftance fhould be always attended to in determining its quantitive proportion in any fubftance that is to be chemically examined.

*On the Separation of the astringent Principle from Vegetables.*

All vegetables, in which the astringent principle has been hitherto acknowledged as a constituent part, possess, besides their astringent effects upon the tongue, the property of tanning the fresh hides of animals, of decomposing most of the metallic solutions, and principally of precipitating the iron from its solution in acids with black colour. Astringent principle, tanning principle, black colouring principle, are therefore terms that have hitherto been considered as synonymous, not only with one another, but also with the Gallic acid, which was first discovered by *Scheele.* — That the astringent and tanning principle, however, is only the same, and that it ought to be well distinguished from Gallic acid, Mr. Seguin * has but lately evinced, by showing that the tanning principle is precipitated from an infusion of oak bark, by the addition of lime-water; whereas the Gallic acid is contained dissolved in the remaining fluid. This fact Mr. Proust † has still more evidently proved, by making known the means of extracting the pure astringent principle separate from the Gallic acid, and by showing that this pure astringent principle is capable of changing in a short time the animal gluten, and even the animal vegetable gluten into a coherent mass, insoluble in water, and resisting putrefaction: and it ought, therefore, on account of acting in the same manner upon animal skins, to be looked upon as the true tanning principle. (Tanin.) According to the same chemist, the method hitherto adopted for obtaining Gallic acid, by precipitating an infusion of galls with a solution of sugar of lead, is by no means the proper way, because the Gallic acid and the astringent principle fall in this manner at the same time to the bottom of the vessel with the calx of lead, which is not the case, when, instead of a solution of lead, a saturated tin in muriatic acid is employed, by which means the astringent matter combined with the calx of tin is precipitated as an insoluble product, while the Gallic acid remains with another part of the calx dissolved in the fluid.

This ingenious discovery leads us to quite a new way of exhibiting separately the astringent principle, in the chemical analysis of astringent vegetables.

For

* Rapport au Comité de salut public, sur les nouveaux moyens de tanner les cuirs proposés par le Citoyen Armand Seguin. Journal des Arts et Manufactures, Paris. An quatrieme, T. ii. p. 66, and T. iii. p. 71, &c.

† Extrait d'un Memoire de Mr. Proust, sur le principe tannant. Annales de Chemie, 25 tom. Paris 1798. p. 225, &c.

For effecting the feparation of the aftringent principle from any vegetable fubftance, this fhould be fufficiently boiled with water and alkohol; and after having mixed both decoctions, the alkohol is firft diftilled over, and the reft evaporated by a gentle fire, till the refiduum becomes dry. This dry mafs muft again be extracted with alkohol, which leaves the gum untouched, diffolving only the faponaceous, refinous, and aftringent principle. To this folution, a faturated folution of muriate of tin, after being diluted with water, is to be poured till it predominates, whereby a precipitation is occafioned. The remaining fluid contains free muriatic acid, muriate of tin, and gallate of tin, along with the other particles of the plant; and after having poured it off, it may be feparately preferved; * but the precipitate is a combination of aftringent principle with the calx of tin. In order to feparate this combination, and to obtain the aftringent principle in a pure ftate, a fufficient quantity of water impregnated with fulphurated hydrogen gas is to be added to the mafs, after it has been diluted with diftilled water. The fulphurated hydrogen, uniting with the calx of tin, precipitates it in the form of a blackifh powder, whereas the aftringent principle remains diffolved, and combined with the fluid. If this is flowly evaporated, the pure aftringent principle is left in the form of a dry and brown fubftance, gloffy when broken in two, whofe weight may alfo be determined.

*On the Separation of the colouring Principle from Vegetables.*

Whether the colouring principle *(principium tingens)* in the vegetables is a principle of its own, or whether it rather originates in the mixture of different matters; and laftly, whether it does not even differ in its elementary mixture according to the difference of colour which it fhows, are queftions that I have not hitherto been able to afcertain, and feveral experiments made on purpofe have ftill left me in doubt on this fubject. What I have found by my own experience is, that in feveral vegetables, the colouring principle is involved with refin, in others with gum and faponaceous matter, and in others

* If the fluid which contains the Gallic acid is impregnated with fulphurated hydrogen gas, the tin falls to the bottom of the veffel, and the Gallic acid remains fluid, and mixed with muriatic acid and the other vegetable fubftances; and if thefe be not in too great a proportion, the Gallic acid may be feparated in a cryftalline form by a flow evaporation; but, on the contrary, if there is too great a quantity of thofe fubftances, it will be impoffible to perform an accurate feparation of it.

with mucilage and farinaceous matter. As this cannot be accurately determined, every fubftance in which the colouring principle is to be difcovered, ought to be expofed to feveral proceffes, as follows:

1. Infufe a plant with diftilled water, and if the colouring principle be combined with gum or faponaceous matter, it will be contained in the infufion, and adhere to a piece of linen, woollen, or filk cloth immerfed in it.

2. Infufe any part of a plant with cauftic fpirit of Ammonia, and expofe it to a gentle digeftion. Should the colouring principle be blended with refin, it will be diffolved, and impart a colour to the fluid, and likewife to linen, woollen, or filk cloth, immerfed in it.

3. If the colouring principle be embodied in mucilage or farinaceous matter, as is the cafe in indigo and madder, a portion of the frefh plant ought to be pounded with water, and after having diluted it with more water, left to ferment; and from the colour of the fcum generating on the furface, it may be concluded whether there is any principle prefent.

I have hitherto not been able to determine its proper quantitive proportion.

### *On the Separation of the cauftic Principle from Vegetables.*

The exiftence of a cauftic principle in vegetables can by no means be denied, but it fufficiently difcovers itfelf in horfe-radifh, in feveral fpecies of creffes, in garlick, by its pungent fmell; and in the arum (wake robin root), and in feveral kinds of cabbage; farther, in Indian and black pepper, and many other vegetables, by its acrimonious tafte. However, I have been taught by numerous operations, inftituted for that purpofe, that it is fcarcely poffible, at leaft by the experiments that have hitherto been made, to feparate it from other combinations, and to afcertain its quantitive proportion in vegetables. This principle being volatile, it may be fomewhat feparated in combination with water, by infufing the plant in which it is contained, and expofing it to diftillation; but this is all that can be faid of it.

I have hitherto endeavoured in vain, by feveral experiments, to exhibit the narcotic and bitter principle of vegetables. The former, however, may at leaft partly be feparated, by treating narcotic vegetables in a retort, without the addition of any fluid, at a temperature of 212° Fahrenheit; whereby a great part of n arcotic matter is obtained in a vapour, that goes over, and is thickened in the cool receiver; the greateft part, however, remains in the retort. But it is by far lefs poffible to reprefent reprefent feparately the bitter principle (principium amarum) of vegetables, and both parts feem to be fo intimately combined with gum, faponaceous matter, and refin, that there is no hope of ever effecting a proper feparation of them, and afcertaining their quantitive proportion.

With refpect to the four or acid falts, as well as to the perfect and imperfect neutral falts and falia media, it is to be obferved, that they are generally contained in vegetables in fuch a fmall quantity, and at the fame time fo intimately united with other component parts, and even intermingled with one another, that it is fcarcely practicable to feparate them entirely from each other, and to determine their quantitive proportion accurately. All, therefore, that I can fay in the following paragraphs about the feparation of thofe fubftances, relates only to the manner of proceeding in general, and I cannot be anfwerable for the refults being always accurate and correct in every fmall inftance.

*On the Separation of Tartarous Acid from Vegetables.*

When upon a previous trial with any vegetable fubftance, the prefence of a free acid is difcovered in it, it is advifable to take the frefh plant for determining and extracting from it the four principle. Any quantity of it is therefore bruifed in a ftone mortar, and the juice expreffed. The refiduum, after being infufed, and ftirred up with diftilled water, is again expreffed, which operation muft be repeated till no free acid is difcovered in it by re-agency. All the juice thus obtained is afterwards boiled up in a tin kettle, or in a glafs veffel, being mixed with the white of eggs, till it is clarified, and its impurities fcummed off. The whole, after having become cool, is filtrated through blotting paper. The clear acidulous juice is then feethed again, and in continually ftirring it, finely powdered oyfter-fhells added, till no effervefcence enfues.— The precipitate, which is hereby caufed, is to be feparated, after having been fuffered to depofite quietly, by cautioufly pouring off the fluid. For feparating from this precipitate the adherent heterogeneous particles, it ought to be repeatedly edulcorated with diftilled water. It is then dryed, and put into eight parts of diftilled water, to which is to be added a quantity of concentrated fulphuric acid, equal to half the weight of the precipitate. This mixture being well ftirred, is digefted for 12 hours, after which time the whole is filtrated, and the refiduum well wafhed out with diftilled water. All the liquor thus obtained, is evaporated by a gentle heat, and by degrees degrees cryſtallized*. The cryſtalline ſalt is the tartarous acid.

### *On the Separation of the Oxalic Acid.*

When a previous examination of a plant diſcovers in it oxalic acid, its juice is after the above mentioned manner to be treated and clarified. The clear liquor is then poured into a diluted ſolution of muriated barytes, by which means the oxalic acid uniting itſelf with the barytes, will fall to the bottom, and the tartarous and malic acid remain. From this precipitate, after it has been carefully waſhed, the oxalic acid may be ſeparated by ſulphur, and exhibited by cryſtallization.

### *On the Separation of the Malic Acid.*

By treating any vegetable juice that contains malic acid in the above manner, the malic acid remains diſſolved and combined with the calcareous earth. In order to ſeparate it, a ſolution of ſugar of lead with diſtilled water ſhould be dropped into that liquor, till no further precipitation is produced. The malic acid falls in this manner to the bottom along with the ſugar of lead, whereas the acetous acid remains combined with the calcareous earth in a diſſolved ſtate. The precipitate being infuſed with diluted ſulphuric acid is committed to a digeſtion, by which means the calx of lead unites with the ſulphuric acid, and the malic acid remains combined with watery particles, from which it may be ſeparated by evaporation.

### *On the Separation of the Benzoic Acid.*

Benzoic acid has been hitherto found in no other vegetable ſubſtance, except reſins and balſams, from which it may be obtained in two different ways.

1. Expoſe any of theſe ſubſtances to a dry diſtillation, whereby the Benzoic acid will partly cryſtallize in the neck of the retort, and partly go over into the receiver with the other particles. For ſeparating it, the neck of the retort is waſhed out with warm water, which is then poured to the reſt that has been diſtilled over; and after having added eight times as much water as what the whole fluid amounts to, it is heated till the

* Should the plant contain at the ſame time oxalic and malic acid, the latter remains diſſolved in the liquor with the calcareous earth at the firſt ſaturation; but the former with the gypſum, as it cannot be ſeparated from the calcareous earth by ſulphuric acid. If there be, however, citric acid preſent in the plant, it remains combined with the tartarous acid, from which it is ſcarcely to be ſeparated.

the water begins to feeth. The hot water receives the Benzoic acid, leaving the oily particles untouched. The whole is feparated by a feparating funnel, and the watery fluid flowly evaporated, till the Benzoic acid begins to cryftallize.

2. A portion of the fubftance that is to be examined is boiled for half an hour with a fourth part of quick lime and fixteen parts of water. The Benzoic acid combines itfelf in this way with the calcareous earth, and diffolves in the water. The liquor being filtrated, is mixed with muriatic acid, till this begins to predominate; by which means the Benzoic acid feparates in fmall cryftals as it cools. The reft of the fluid, when evaporated, affords ftill more of the acid.

*On the Separation of the Saccholactic Acid.*

The faccholactic acid has not been hitherto determined to be a component part in vegetables, but the gummy and mucilaginous matters always yield it when they are boiled with nitrous acid. Whether, therefore, it is really a component part of thofe fubftances, or whether it is firft compofed during the operation from them and the oxygen of the nitrous acid, has not yet been fufficiently afcertained. This acid may be obtained from thofe fubftances, by boiling for five minutes one part of them with eight parts of nitrous acid, (confifting of one part of fpiritus nitrifumans and one part and a half of water). After the refiduum becomes cool, the faccholactic feparates in form of an acidulated difficultly foluble powder.

*On the Separation of Tartar and Salt of Sorrel.*

When an effential four falt, either tartar or falt of forrel, or both mixed together, is traced in any plant, the feparation fucceeds beft in the following manner.

1. A certain quantity of the frefh plant is expreffed, and the juice clarified after the above method. This juice being thickened to the confiftency of a fyrup, is infufed with fix parts of rectified fpirit of wine, and digefted; whereby, if any of thofe exifted in the plants, it is found cryftallized after the fluid becomes cool.

2. The cryftallized falt fhould then be feparated from the fluid, and by being repeatedly diffolved in diftilled water, difengaged from any adherent heterogeneous particle.

3. The form of the cryftals fhows whether it is pure tartar or pure falt of forrel; but if both are mixed with each other, this may be difcovered in the following manner: Put a little into a filver fpoon, and hold it over hot coals. Should the mafs then emit during the melting, vapours fmelling like burned burned ſugar, without forming a ſublimate, on a glaſs plate held over it, it is pure tartar; but if white vapours riſe on a glaſs plate to a white and ſour powder, this is the oxalic acid, which ſhows the mixture of ſalt contained to be ſalt of ſorrel. *

*On the Separation of Saltpetre from Vegetables.*

When a plant contains ſaltpetre, this is eaſily diſcovered by burning the dry plant in a candle; whereby, if but little ſaltpetre exiſts in it, a weak detonation is remarked. Being in this manner convinced of the preſence of ſaltpetre, bruiſe a certain quantity of the freſh plant, and expreſs the juice, which having been clarified according to the above method, and thickened to the conſiſtency of a ſyrup, is alſo treated with rectified ſpirit of wine, whereby the ſaltpetre is obtained in a cryſtalline form, after the ſpirituous digeſtion being cooled.

[ To be continued. ]

* When the vegetable acids are perfectly neutralized in plants with alkalies or earths, and principally when theſe combinations are eaſily ſoluble, they are ſo intimately combined with ſaponaceous matter, that a ſeparation is in vain expected, and one muſt be contented with only knowing their exiſtence in vegetables. The neutralization with ammonia is recognized, by adding quick-lime, from the volatile ſmell, and the white vapours that riſe, when a piece of wood, moiſtened with concentrated acetic acid, is held over it. A ſeparation of the ammoniacal neutral ſalts is likewiſe not obtainable, even if the neutralization be effected by mineral acids.

CRITICAL

